# The Immune Response of Cancer Cells in Breast and Gynecologic Neoplasms

**DOI:** 10.3390/ijms25116206

**Published:** 2024-06-05

**Authors:** Katarzyna Rakoczy, Justyna Kaczor, Adam Sołtyk, Natalia Szymańska, Jakub Stecko, Małgorzata Drąg-Zalesińska, Julita Kulbacka

**Affiliations:** 1 Faculty of Medicine, Wroclaw Medical University, Pasteura 1, 50-367 Wroclaw, Poland; katarzyna.rakoczy@student.umw.edu.pl (K.R.); justyna.kaczor@student.umw.edu.pl (J.K.); adam.soltyk@student.umw.edu.pl (A.S.); natalia.szymanska@student.umw.edu.pl (N.S.); jakub.stecko@student.umw.edu.pl (J.S.); 2 Department of Human Morphology and Embryology, Division of Histology and Embryology, Faculty of Medicine, Wroclaw Medical University, T. Chalubińskiego 6a, 50-368 Wroclaw, Poland; malgorzata.drag-zalesinska@umw.edu.pl; 3 Department of Molecular and Cellular Biology, Faculty of Pharmacy, Wroclaw Medical University, Borowska 211a, 50-556 Wroclaw, Poland; 4 Department of Immunology and Bioelectrochemistry, State Research Institute Centre for Innovative Medicine Santariškių g. 5, LT-08406 Vilnius, Lithuania

**Keywords:** immune response, gynecological cancers, breast cancer, tumor microenvironment

## Abstract

Cancer diseases constitute a major health problem which leads to the death of millions of people annually. They are unique among other diseases because cancer cells can perfectly adapt to the environment that they create themselves. This environment is usually highly hostile and for normal cells it would be hugely difficult to survive, however neoplastic cells not only can survive but also manage to proliferate. One of the reasons is that they can alter immunological pathways which allow them to be flexible and change their phenotype to the one needed in specific conditions. The aim of this paper is to describe some of these immunological pathways that play significant roles in gynecologic neoplasms as well as review recent research in this field. It is of high importance to possess extensive knowledge about these processes, as greater understanding leads to creating more specialized therapies which may prove highly effective in the future.

## 1. Introduction

Neoplastic diseases are an obstacle on medicine’s path to extend life expectancy, as they constitute an increasingly leading cause of human deaths. In the single year 2020, an estimated 19.3 million new cases arose worldwide, leading to nearly 10 million cancer deaths [1,2]. Among them, more than 3.6 million cases were breast cancer and gynecological cancers, including ovarian cancer, vulvar cancer, vaginal cancer, cervical cancer, and uterine cancer. They led to over 1.3 million cancer deaths amongst women, whereas the most lethal one was ovarian cancer, in the case of which approximately 66% of cases resulted in death [1]. Cancer cells are extraordinary in every possible biological aspect, which made them stand in the center of neoplastic attention for decades. For instance, while under aerobic conditions normal host cells acquire energy via oxidative phosphorylation in mitochondria and reprogram themselves to rely on glycolysis when the amount of oxygen decreases, cancer cells prefer glycolysis to oxidative phosphorylation even with access to oxygen due to faster, though less effective, ATP supply [3,4]. The described phenomenon is called the Warburg effect. Even though described metabolic alteration is the most widely known, it is definitely not the only one that differentiates metabolism of cancer cells from that of normal host cells. Neoplastic cells are capable of suppressing anti-tumor immune response as they deprive immune cells of nutrients [5,6]. What is more, metabolites present in the surroundings of the tumor influence differentiation and functioning of those cells and thus, cancer immune response and immune cells metabolic reprograming seem to intertwine in their semantic dimensions [7,8]. For this reason, in the past few years, more and more attention has been drawn to non-cancerous cells present in tumors, mainly immune cells, which turn out to play significant roles in the course of the disease. Thus, neoplastic diseases became regarded as dynamical, evolutionary phenomena characterized by the multitude and multidimensionality of interactions between cancer cells and their surroundings, created by extracellular matrix (ECM), a variety of dissolved molecules, such as chemokines, as well as all types of non-cancerous cells that can be found in the vicinity of the neoplasm. Enumerated factors altogether create dynamically changing space, also known as the tumor microenvironment (TME) [9,10]. TME must therefore not be understood as a random collection of cellular and non-cellular components that happen to surround cancer cells. On the contrary, in the light of the most recent research, it constitutes complex creation, the contribution of which, in varying degrees, determines cancer cells’ ability to avoid immune response and cell death, as well as angiogenesis and metastasis [11]. The importance of the role that TME plays in the survival of neoplasm makes its elements and signaling pathways that link them a promising targets of cancer therapies. Therapies, which on the one hand might prove more effective than the ones targeting cancer cells, as non-neoplastic cells, thanks to their genetic stability, are not vulnerable to drug resistance and thus are much more vulnerable to drugs themselves. On the other hand, it must be emphasized that non-neoplastic cells present in TME do not only exist in TME but usually have a multitude of important functions outside the tumor surroundings [12]. For this reason, it is crucial to find and understand the molecular differences between normal non-neoplastic cells and non-neoplastic cells present in TME. Only in this way targeting TME would not equal targeting all host cells of certain cell types and thus provoking disastrous adverse effects.

In this review, we summarize recent knowledge about the most significant types of non-neoplastic cells present in TME as well as describe the most important pro-tumoral pathways and discuss potential molecular therapy targets that can be found inside of their complexity, focusing our attention on breast and gynecologic neoplasms. 

## 2. Tumor Microenvironment 

**Figure 1:**
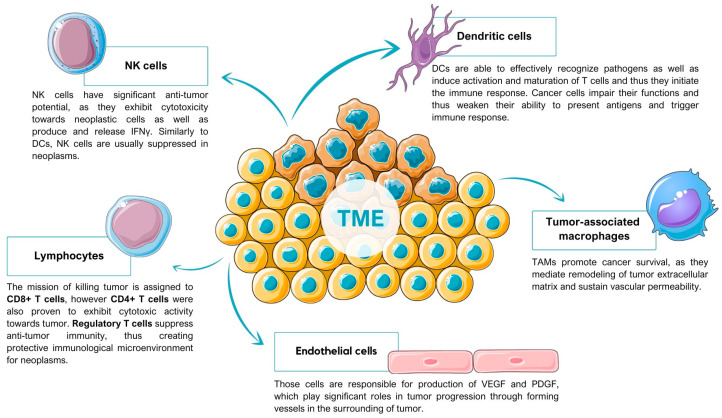
Schematic visualization of selected types of cells that are present in tumor microenvironment and their properties [13,14,15,16,17].

Neoplasms constitute the most rapidly evolving type of disease, the dynamic state of which is sustained by continual, differentiated interactions between cancer cells and elements of TME. Those elements consist of non-cancerous cells as well as non-cellular components that happen to create the surrounding of the tumor, inter alia endothelial cells, adaptive and innate immune cells, chemokines, cytokines, and extracellular vesicles (Figure 1). 

The most characteristic property of TME is definitely its multidimensional complexity, which is not only based on the multitude of factors that create it, but also on the phenomenon of increased heterogeneity of its cellular components. As it is widely known, the surrounding of the tumor is unlike any other microenvironment in the human organism, as it abounds in metabolic anomalies that include low nutrient supply, hypoxia, inflammation, and many more. For this reason, the influence of the environmental conditions on immune cells is unavoidable and constitutes the fundament of their heterogeneity in tumors. Research has proven that immune cells in TME present more phenotypes than the ones in healthy tissues. For instance, in human breast cancer (BC) tissue 17 differentiated clusters of T cells were found, whereas only half of them were present in non-neoplastic breast tissue [18]. The uniqueness and significance of TME makes researchers target its components in therapies, which brings more and more promising effects. As can be seen when it comes to BC treatment, a variety of novel drugs are currently in preclinical and clinical studies (Table 1).

The tumor microenvironment is continually proving to play a vital role in gynecological cancers. Therefore, let us consider its impact on pathogenesis and the development of two neoplastic diseases—inflammatory breast cancer (IBC), the most lethal and the least treatable of BCs, and ovarian cancer (OC). As numerous studies suggest, TMEs of both IBC and OC are characterized by an inimitable combination of cell types. The balance between chronic inflammatory pathways and healthy immune defense mechanisms is distorted in favor of the first enumerated components [14,28]. Let us analyze the most impactful of cells present in TME of the discussed cancers in order to understand the complexity of interactions that shape the characteristics of IBC and OC. 

### 2.1. Tumor-Associated Macrophages

Tumor-associated macrophages (TAMs), depending on the local tumor microenvironment, can differentiate into two separate phenotypes, M1 and M2. The M1 phenotype is known as the pro-inflammatory one, which exhibits anti-tumor activity, whereas the M2 phenotype constitutes an anti-inflammatory agent that promotes tumor progression [29]. TAMs were proven to promote cancer survival, as they mediate remodeling of tumor extracellular matrix as well as sustain vascular permeability [30]. When it comes to IBC, research conducted in vitro revealed that IL-6 secretion from epithelial cells, intermediated in the IBC-promoting effect of TAMs and thus implementing IL-6 antibodies, could lessen the impact of TAMs on IBC cells [31]. In vivo studies, on the other hand, pointed out that targeting macrophage colony stimulating factor 1 (CSF1), which participates in polarization of M2 macrophages, harnesses the IBC aggressive phenotype [31]. Regarding OC, it has been proven that TAMs that overexpress sialic acid–binding Ig-like lectin 10 (Si-glec-10) interact with CD24 expressed by the tumor and thus promote immune invasion [32]. What is more, in mouse models of OC, TAMs required specific protein zinc finger E-box-binding homeobox 1 (ZEB1), for their tumor-promoting activity and TAMs with full ZEB1 gene expression turned out to enhance tumor growth as ZEB1 induces polarization of TAMs in the pro-tumor direction. In human OC, CCR2 expression alongside with TAM infiltration correlated with ZEB1 in tumor cells, leading to poorer prognosis. ZEB1 in TAMs proved to be a factor of lower survival rate. Thus, ZEB1 appears to play an indirect yet vital role in TME as it preserves cancer-promoting function of TAMs [17]. Optimistically though, research suggest that M2-like polarization of TAMs can be suppressed by sorbin and SH3 domain containing 2 (SORBS2) and thus metastatic colonization of OC can be repressed [33]. 

### 2.2. Dendritic Cells 

Dendritic cells (DCs) constitute quintessential antigen-presenting cells (APCs), which constitute the cellular bridge between innate and adaptive immunity. Described cells originate from common myeloid progenitors (CMPs) localized in the bone marrow and divided into two cell types. Under inflammatory conditions, expression of Nur77 transcription factor promotes CMP differentiation into monocyte DCs (moDCs), whereas under the conditions of Nur77 absence, progenitors differentiate into the common dendritic cell progenitors (CDPs), from which two populations, plasmacytoid DCs (pDCs) and predendritic cells (pre-mDCs) arise. Pre-mDCs constitute a common precursor of myeloid DCs (mDCs) [13]. Through Toll-like receptors (TLR), DCs are able to recognize effectively pathogens as well as induce activation and maturation of T cells and thus, they initiate the immune response [15]. In patients with IBC, measuring DC count in peripheral blood allows their function to be determined. One such study revealed lower mDC count in patients with IBC in comparison to healthy donors. Combining DC count with circulating tumor cell (CTC) count, on the other hand, proved that patients with IBC with a high number of CTCs showed poorer clinical outcomes than patients with a low number of CTCs and a high number of TLR-activated mDCs. Moreover, poorer clinical outcome was also proven to be connected with expression of CC-chemokine receptor 7 (CCR7) and CD86 on TLR-activated DCs, positively associated with CTC count [34].

Ovarian cancer cells are able to impair the function of DCs and thus weaken their ability to present antigens and trigger immune response. In the draining lymphatic nodes, mDCs increase programmed death ligand 1 (PD-L1) expression and, therefore, are not able to cause activation of T cells [35]. What is more, research has shown that in solid cancers the presence of transforming growth factor-β (TGFβ) DCs are unable to promote T cell proliferation, as TGFβ encourages CD4+ T cell differentiation towards CD4+CD25+FOXP3+ Treg cells [36]. Described conclusions from the research suggest that OC immunotherapy based on DCs is within our reach. One such study proved that autologous dendritic cell immunotherapy consisting of dendritic cells for ovarian cancer (DCVAC/OvCA), which can present tumor antigens and thus provoke a durable immune response in patients with epithelial ovarian cancer (EOC), improves cancer prognosis. In patients undergoing first-line treatment of EOC, a statistically noteworthy effect of DCVAC/OvCA administered successively with chemotherapy was found. In comparison to chemotherapy itself, the combination of the described two lines of therapy delayed progression of EOC [37].

### 2.3. T Cells

T cells are of invaluable importance for tumor immunity as T cell receptors (TCRs) recognize tumor antigens, which leads to release of perforin and granzymes and thus apoptosis of tumor cells. For this reason, their activity is enhanced by major therapeutics, which include PD-1 antibodies that function as a blockade for inhibitory T cell receptors, as well as genetically engineered T cells, which express chimeric antigen receptors (CARs) specific towards the tumor [38,39]. Importantly, even though the mission of killing a tumor is assigned to CD8+ T cells, in recent studies CD4+ T cells were proven to exhibit cytotoxic activity towards a tumor as well [40]. Among the subpopulations of T cells, regulatory T (T reg) cells are also worth mentioning, as they suppress anti-tumor immunity, thus creating protective immunological microenvironment for neoplasms. Although, seemingly, therapies targeting this subpopulation of T cells should result in increased anti-tumor activity, the fact that T reg cells are essential for immune tolerance of the whole organism as well as constituting an important part of molecular signaling pathways cannot be omitted. For this reason, as long as we are unable to target specifically T reg cells present in TME without impairing the function of other T reg cells, consideration of this subpopulation as a novel therapeutic approach remains a challenge [16]. IBC and non-IBC patients differ in multiple ways when it comes to the functioning of T lymphocyte cells. First of all, overall lymphocyte count in peripheral blood is significantly lower in patients with metastatic IBC than in non-IBC patients [34]. IBC patients also exhibit lower counts of T cell receptor (TCR) activated CD4+ T cells, producing IL-4 as well as TCR-activated CD8+ T cells, which release IL-10 [41]. Another important factor that differentiates IBC and non-IBC patients is PD-L1 expression. Overexpression of the ligand is found in more than one third of samples and is directly connected with aggressive subtypes and constitutes an independent predictive value for chemotherapy pathological response. From the therapeutical perspective, inhibiting PD-L1 with the PD-L1 antibody atezolizumab, should enhance immune response to treatment, as it should shield activated T cells as well as reactivate inhibited T cells [35,42]. The effects of therapy are most notable in combination with immunogenic chemotherapy [43]. Blockade of PD-L1 or programmed cell death protein 1 (PD-1) offers similar results. Interestingly, immunotherapy also presents promising new strategies for treatment in uterine cancer, particularly for advanced cases where traditional therapies fall short. Immunomodulation of TME, including active and passive immunotherapy, is being actively explored as a means to enhance treatment efficacy and provide durable responses. Current clinical trials and studies, such as those involving pembrolizumab and dostarlimab, demonstrate the potential of these therapies in improving outcomes for patients with endometrial cancer [44,45]. However, in many patients the response to checkpoint inhibitor therapies is too weak or untraceable, which motivates the search for a combination with another therapeutic approach, that might turn out to be efficient. For this reason, one of the studies of combined therapy described above shows that C-X-C chemokine receptor type 4 (CXCR4) inhibition with plerixafor is able to decrease T reg infiltration. In vivo studies exhibited that the immunomodulating potential of two combined therapies is significant, as its application increased effector T cell infiltration, decreased intra-tumoral T reg cell count, as well as promoted T reg cell differentiation towards T helper cells. Apart from the influence on T cells, therapy also resulted in enhanced M2 macrophage polarization towards M1 macrophages [46].

When it comes to reawakening T cells, research on the impact of epidermal growth factor receptor (EGFR) cannot be omitted. EGFR plays the key role when it comes to immunosuppressive TME maintenance in IBC, as it constitutes one of the main signaling molecules in TME. Inhibiting EGFR with panitumumab, a humanized antibody, regulates expression of chemokines in IBC cells and thus modifies TME. In vivo, altered expression of chemokines results in recruitment of CD8+ T immune cells as well as lowered infiltration of T reg cells and M2 macrophages [47]. In OC patients, high concentration of FOXP3+ Treg cells is connected with increased cancer mortality. These lymphoid cells are recruited by hypoxia-induced CCL28 and CCL22 and promote an immune microenvironment which supports tumor growth [48,49]. Suppressive capacity of T reg cells increases when the cells become apoptotic. Apoptosis of T reg cells in TME is observed under oxidative stress conditions [50]. Apoptotic T reg cells, through CD39 and CD73, convert ATP to adenosine and arbitrate PD-L1 inhibitor resistance. Simultaneously, CD8+ T cells infiltrate into the tumor [51]. Heterogeneity of immune TME expresses itself in the fact that despite observed influx of CD8+ T cells, recruited lymphoid cells are usually dysfunctional. As the research has proven, only 10% of CD8+ T cells in TME are able to recognize autologous OC cells [51]. This phenomenon can be explained by the fact that in the tumor surrounding, where antigen exposure is constant, T cells might consequently weaken their functions over time. During that period, T lymphocytes upregulate expression of inhibitory receptors, such as PD-1. However, the described vegetative state of T cells can be turned upside-down by inhibitory receptor inhibitors, for instance PD-1 antibody [52]. What is more, TME is characterized by nutrient-poor, glucose-deprived conditions, which impedes mitochondrial respiration and IFNγ as well as other intermediates production by T cells [53]. 

### 2.4. Natural Killer Cells

Natural killer (NK) cells have significant anti-tumor potential, as they exhibit cytotoxicity towards neoplastic cells as well as produce and release IFNγ. Similar to DCs, NK cells are usually suppressed in TME of IBC. When it comes to OC, recent research suggests that molecules present in TME of OC exhibit the capability to control activity of NK cells. Thus, they cause NK cells to overexpress PD-1. As a consequence, anti-tumor activity of the cells decreases, as they exhibit weakened cytotoxic potential as well as lower production and release of cytokines [54]. As it can be deducted, PD-1 inhibitors once again turn out to be of great usability, reawakening NK cell anti-cancer properties. What is more, research proved that NK cells delivered from induced pluripotent stem cells (iPSCs) expressed both characteristic markers of NK cells and CARs, which enhanced their anti-tumor activity. In an OC patient-derived xenograft murine model, human NK-CAR iPSC-NK cells turned out to retard tumor progression, as they selectively targeted OC cells. Therefore, the CAR-NK therapeutical approach seems to forge a novel path for OC treatment [55,56]. 

### 2.5. Endothelial Cells 

Endothelial cells are responsible for production and release of vascular endothelial growth factor (VEGF) as well as platelet-derived growth factor (PDGF), which play significant roles in tumor progression through forming vessels in the surrounding of both IBC and OC cells. IBC cells, in comparison to non-tumor cells, overexpress VEGF-D, which alongside VEGF-C activates VEGF receptor 3. VEGF-D was proven to play a crucial role in angiogenesis and lymphangiogenesis of IBC [57,58,59]. As expected, infiltration of lymphatic endothelial cells in IBC is usually significant. Regarding OC cells, research in vivo has shown that expression of Notch 1 intracellular domain (N1ICD) in endothelial cells of OC supports peritoneal metastasis in mice, thus leading to lower survival rates. Activated N1ICD is conductive to vascular cell adhesion molecule 1 (VCAM1) expression, recruitment of neutrophils, and intravasation of the tumor [60]. Another important molecular factor that influences OC is the family of miR-200, among which miR-200c plays a crucial role, as it regulates angiogenesis negatively. During in vitro studies, overexpression of miR-200c led to decreased expression of IL-8 and CXCL1 secreted by cancer cells as well as tumor-associated endothelial cells. The decrease could be reversed by miR-200c inhibitors, which confirms their role in restraining angiogenesis [61]. Another important miRNA connected with OC is miR-145, which downregulates the level of VEGF, thus suppressing angiogenesis and tumor growth. The described miRNAs could be used as novel therapeutical approaches [62].

## 3. Pro-Tumoral Pathways

### 3.1. STAT3

**Figure 2:**
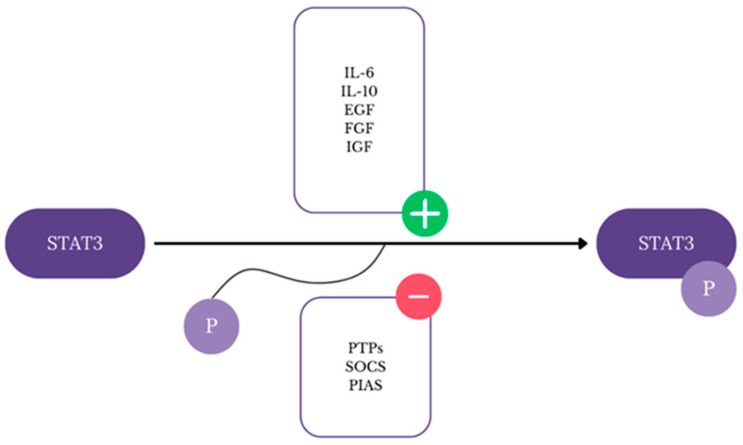
Visualization of positive and negative regulators of STAT3 [63,64,65,66].

STAT3 (signal transducer and activator of transcription 3) is a member of the STAT family that plays a crucial role in the regulation of the cellular process [63]. As it has a great impact on cell functions, it comes under strict regulations and there are several factors that can activate STAT3 including interleukin 6 (IL-6), interleukin 10 (IL-10), epidermal growth factor (EGF), fibroblast growth factor (FGF), and insulin-like growth factor (IGF) [63,64]. When the previously mentioned positive regulators bind to their receptors, STAT3 molecules are phosphorylated by tyrosine kinases such as JAK (Figure 2) [65]. Activated STAT3 is translocated to the nucleus, binds to its target gene, and regulates its transcription [63,65]. The malfunction of the JAK/STAT3 signaling pathway is associated with cancer development and may be a promising target for new treatment; thus, understanding the role of JAK/STAT3 inhibitors may be crucial to achieve a breakthrough in cancer therapy [65,66,67]. 

**Figure 3:**
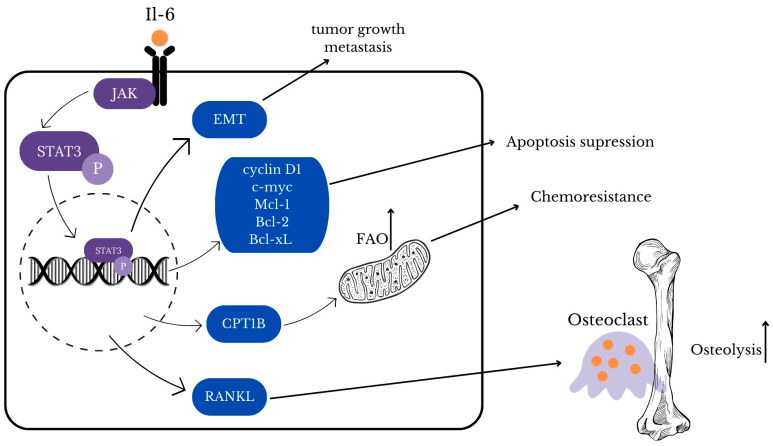
Visualization of the complicity of STAT-3 pathways activated via Il-6 [63,64,65,66,67,68,69,70,71,72,73,74].

Among negative regulators there are protein tyrosine phosphatases (PTPs), suppressor of cytokine signaling (SOCS) proteins, and protein inhibitors of activated STAT (PIAS) that inhibit STAT3 through blocking phosphorylation or DNA-binding [65,66]. In cancer cells, negative regulators are inhibited, so the STAT3 pathway remains active which results in cancer progression [65]. Breast, ovarian, and cervical cancer, as many other cancers, present elevated IL-6/JAK/STAT3 pathway activity [68]. STAT3 activation promotes proliferation and suppression of apoptosis of breast tumor cells through the upregulation of target genes cyclin D1, c-myc, Mcl-1, Bcl-2, and Bcl-xL [64,68]. JAK2/STAT3 signaling pathway takes part in the development of chemoresistance in breast cancer by increasing carnitine palmitoyl transferase 1B (CPT1B) and fatty acid beta-oxidation (FAO) [64]. Breast cancer is characterized by increased IL-6 levels which is suspected to be a result of single nucleotide polymorphisms (SNPs) and is associated with poor prognosis in patients [68]. Moreover, both IL-6 and IL-11 induce the development of bone metastases through various pathways [69]. IL-11 through STAT3 activation stimulates osteoblast activity, which is dominant in the metastasis of breast cancer cells to the bone [68,70]. Both IL-6 and IL-11 via STAT3 phosphorylation promote receptor activator of nuclear factor kappa B ligand (RANKL) expression that stimulates differentiation of osteoclasts [69,70]. In the study of Liang M. et al. in vitro and in vivo blockade of STAT3 phosphorylation inhibit osteolysis and metastatic dissemination to the bones [70,71]. IL-6 can act as an inflammatory factor which mediates epithelial-to-mesenchymal transition (EMT), a process that promotes tumor growth and metastasis [69]. IL-6/JAK/STAT3 pathway leads to EMT activation not only in breast cancer but also in cervical carcinoma (Figure 3) [68,69]. Oncogenic human papillomavirus (HPV) infection is associated with about 4.5% of cancers worldwide including mainly cervical carcinomas but also cancers in the anogenital and head and neck regions [72]. HPV cell cycle is regulated by STAT3 and loss of active STAT3 leads to blockade of cell cycle progression and abolishment of virus genome amplification especially in HPV18 [73]. In conclusion, connection between STAT3 and HPV life cycle plays an important role in cervical cancer developing. STAT3 regulates autophagy that provides tumor cells with nutrients and plays certain roles in drug resistance, but may lead to cell death when exaggerated [74]. In the study of Wu L. et al., it was proved that STAT3 through the Bcl2-Beclin1 axis can increase the level of autophagy in cancer cells [74,75]. Additionally, STAT3-induced autophagy provides a survival advantage to cancer cells under stress conditions, contributing to chemoresistance [76]. Considering current clinical studies, inhibitors targeting STAT3, such as WP1066, are being investigated for their potential to reverse chemoresistance and improve therapeutic outcomes in ovarian cancer, which seems promising in future treatments [77]. 

### 3.2. Annexin 1

Annexin A1 (ANXA1), commonly referred to as lipocortin-1 or renocortin, is a 37-kDa (346 amino acids) member of the annexin superfamily of Ca^2+^ and phospholipid binding proteins contributing to its involvement in membrane-related events [78]. Until recently, it was believed that its main role was as a second messenger (mediator) in glucocorticoid anti-inflammatory action as an inhibitor of phospholipase A2 activity [79]. ANXA1 has been shown to regulate immune response, cell proliferation, and apoptosis [80,81]. 

As we currently know, chronic inflammation promotes not only obesity, cardiovascular diseases, diabetes mellitus but also tumoral pathways. Inflammation is a very complex process; broadly accessible parameters, e.g., CRP do not describe it sufficiently and more subtle parameters are essential. M. van Erk et al. found that ANXA1 expression was induced after 9 days administration of diclofenac, non-steroidal anti-inflammatory drug (NSAID), which in turn inhibited A2 and COX-2 enzymes, thereby inhibiting prostaglandin synthesis [82]. Passive immunization with anty-ANXA1 antibodies abolishes the anti-inflammatory effect of administered dexamethasone. In summary ANXA1 has anti-inflammatory and anti-migrating properties [80,83]. ANXA1 can be detected in extracellular vesicles in blood serum from patients with inflammatory bowel disease but not in serum from healthy donors [84]. ANXA1 was found in many cells and tissues (lung, bone, marrow, intestine, peripheral blood T-cells, neutrophils, placenta, liver) [80,85,86,87]. It is overexpressed in the ectopic endometrium of women with endometriosis and presents in the peritoneal fluids of patients with endometriosis [81]. Human expression data indicate a correlation between immune infiltration and overall ANXA1 expression in malignant compared to healthy tissue [88]. Meta-analysis performed by Z.M Tang et al. proves ANXA1 antibodies are a prominent tool to diagnose lung cancer in all stages, although individual tumor-associated autoantibodies (TAAbs) like ANXA1 showed low diagnostic sensitivity and the combination of multiplex autoantibodies ANXA1 alongside p53, NY-ESO-1, CAGE, GBU4-5, SOX2 offered relatively high sensitivity [89]. The strong relationship between ANXA1 and cancers was investigated by D. Zhu et al. ANXA1 expression correlated to a significant grade in OSCC patients; lower ANXA1 expression was correlated with a poorer differentiation grade; ANXA1 overexpression reduced the cell proliferation while its down-regulation increased proliferation of HB96 cells [90]. ANXA1 high levels on the contrary to BC, melanoma, pancreatic cancer, are low or undetectable in other tumors, e.g., esophageal, head and neck, prostate cancer, oral squamous cell carcinoma, or even show different levels of GlcNAcylation (colorectal cancer) [90,91,92,93]. The inconsistent data may seem absurd, but perhaps an explanation of this contradiction exists. One such explanation is based on the fact that ANXA1 can be specific to each tumor type due to post-translational modifications of the protein, which could account for the alterations in surface expression seen on different cells and in different cancers [94]. ANXA1 is known for anti-proliferative and proapoptotic properties [95,96]. ANXA1 mediates proliferation by intercepting with EGF and EGFR tyrosine kinase and induces apoptosis through two mechanisms: its overexpression followed by dephosphorization of the BAD or of its recruitment the cell surface, where it binds with phosphatidylserine [80,87]. Cancers tend to get rid of substances like ANXA1 through down regulation so its development is not intercepted [97]. Loosing ANXA1 may lead to cancer cell resistance to apoptosis induced by chemotherapeutic agents [80]. A different point of view is provides by T. Hein et al.: their work suggests that the annexin family proteins (ANXA1 included) migrate towards the outer leaflet of the plasma membrane during the end of apoptosis (or in the early stage), triggering peripheral immune tolerance of cancer antigens via Dectin-1 receptor [88]. It would mean expressing ANXA1 and is beneficial for cancer cells, which makes it a potential target for anti-cancer therapy and the explanation of emerging drug resistance of cancer cells after treatment. This effect was observed for many tumors, e.g., breast adenocarcinoma. In the 2004 group from California, USA, they obtained positive results after administration of monoclonal ANXA1 antibodies combined with radiotherapy against a rat tumor model. Low levels of radionuclides (100 mCi) were delivered. The outcome achieved was that tumor was destroyed, which increased animal survival [98]. Another animal study used ANXA1 KO mice to show that ANXA1 is the main regulator of balance between physiological and pathological balance. When ANXA1 is absent, tumors grow slower, form fewer blood vessels, and fail to metastasize, which elongates animals lives [99]. It may seem a promising experiment, however, how can we mop ANXA1 up from human cells without intervention in the genome? Nevertheless, we could use ANXA1 as an angiogenesis marker. Likewise, pancreatic cancer progression and metastasis are promoted by ANXA1 via micro vessel development, cytoskeletal remodeling leading to cell motility, and as an agonist of formyl peptide receptors [100]. ANXA1 plays a significant role in the inflammatory response and has been implicated in the progression of several gynecological malignancies, including endometrial and ovarian cancers. The available epidemiological studies have shown that elevated levels of ANXA1 correlate with poor prognosis and advanced disease stages in these cancers [101,102]. Although specific clinical trials targeting ANXA1 are limited, its involvement in cancer progression highlights its potential as a therapeutic target. ANXA1 is implicated in the regulation of both apoptosis and autophagy in cancer cells. In gynecological cancers, ANXA1 promotes tumor progression by inhibiting apoptosis and facilitating autophagy, thus providing cancer cells with mechanisms to survive under adverse conditions [102]. What about BC? ANXA1 modulates human epidermal growth factor receptor 2 (HER2)-positive BC response to trastuzumab. Tumors with low level of ANXA1 showed a benefit from trastuzumab, while no effect was detected in tumors with high expression of ANXA1 [103]. ANXA1 was not found to be related to tumor response to chemotherapy (doxorubicin + docetaxel or doxorubicin + paclitaxel) [104]. BC is associated with normal or elevated levels of ANXA1; however, some tumors tend to lose it, which might suggest its importance in maintaining normal breast biology as a negative regulator of cancer cell growth [105]. Another study involved stimulating MCF-7 breast cancer cells with various estrogen levels and exposure of physiologic estrogen levels led to upregulation of ANXA1 [95]. The authors suggest that ANXA1 may act as a tumor suppressor gene capable of modulating the proliferative function of estrogens. ANXA1 expression varies in different types of breast cancer (Table 2).

### 3.3. CD47/SIRPα

The CD47/SIRPα axis has been known since 1998 and its role in neoplasms cannot go unnoticed [113,114]. It enables cancer cells to avoid both being detected by macrophages and phagocytized [115]. Therefore, blocking this axis presents a potential in therapies of different types of cancer since it makes the tumor much more vulnerable to innate and adaptive immune response. However, in order to fully understand the implication that it might present clinically, first, we shall proceed with the characteristics of this pathway. 

#### 3.3.1. Characteristics of CD47-SIRPα Axis

Cluster of differentiation 47 (CD47) is the transmembrane glycoprotein widely expressed in all types of cells. It contains one Ig-like domain located extracellularly and five transmembrane domains [115]. Contrarily, SIRPα, signal regulatory protein α, can be found only in macrophages, granulocytes, monocytes, dendritic cells, and neurons and its levels of expression vary [114,116]. Extracellularly SIRPα contains three Ig-like domains and intracellularly two typical immunoreceptor tyrosine-based inhibitory motifs—ITIMs [115]. The latter ones initiate an inhibitory signal when the two Ig-like domains—one of SIRPα and the other of CD47—recognize each other. Such interaction between these molecules initiates a phosphorylation of SIRPα ITIMs, therefore leading to the repression of phagocytosis. That is known as “don’t eat me signal” [117]. In macrophages this result is performed via suppression of myosin IIA—consequently being a critical step in blocking phagocytosis [115]. CD47/SIRPα plays its part not only in cancer but also is crucial for red blood cell maintenance under physiological conditions. A suchlike correlation was found in 2000 when the CD47 was proved to be a marker for red blood cells and when interaction with SIRPα occurred, the phagocytosis by red pulp macrophages was inhibited [118]. Its interaction with the signal regulatory protein alpha (SIRPα) on macrophages inhibits apoptosis and supports tumor cell survival. Blocking CD47 with specific antibodies can enhance the phagocytosis of cancer cells and is being investigated in combination with autophagy inhibitors to induce cancer cell death more effectively [119]. CD47/SIRPα axis is upregulated as well in circulating hematopoietic stem cells and progenitor cells [115,120]. Given that certain challenges may arise when it comes to using anti-CD47 antibodies in cancer immunotherapy as apart from blocking the protein expressed on cancer cells, CD47 on non-cancerous cells including red blood cells will be blocked as well. That leads to an enhanced sequestration of RBCs by the reticuloendothelial system and therefore favors splenomegaly [121,122].

#### 3.3.2. CD47/SIRPA Axis in Neoplasms 

It has been discovered that a variety of cancer cells present high expression level of CD47 which correlates with poor prognosis of the disease. CD47 is overexpressed in various gynecological cancers such as ovarian and endometrial cancers, contributing to immune evasion by inhibiting macrophage phagocytosis of cancer cells [123]. Especially in regard to ovarian cancer, squamous cell carcinoma of the head and neck, gliomas, osteosarcoma, and melanoma [124,125,126,127,128]. Overexpression of CD47 is also stated in acute myeloid leukemia stem cells as well as in patients with glioblastoma, ovarian, breast, bladder, colon, and hepatocellular cancer [117]. Expression of CD47 is regulated by a variety of factors such as Myc oncogene that stimulate expression of CD47 gene, which has been studied in models of T cell acute lymphoblastic leukemia (T-ALL) and in oral squamous cell carcinoma (OSCC) [129,130]. TNFα is an example of an extracellular stimulator that activates NFκB (nuclear factor-κB) to bind to an enhancer of CD47, thus resulting in increased gene expression of this molecule [117]. Such interaction has been observed in MCF-7 breast cancer and inhibition of phagocytosis resulted in tumor growth [131]. Hypoxia is another factor that is not indifferent to cancer cells [117]. Analysis of datasets derived from thousands of breast cancer patients unveiled the connection between the expression of HIF-1 (hypoxia-inducible factor), CD47, and increased mortality of patients [18]. HIF-1 increases CD47 expression by directly activating transcription of its gene in hypoxic breast cancer cells which consequently leads to inhibition of phagocytosis [117,132]. Additionally, CD47 may be also observed on exosomes [133,134,135], vesicles actively secreted by cells that effectively enter into other cells [136]. It has been noted that CD47 in high levels on the exosomes of breast cancer patients may be unfavorable when it comes to the course of the disease [133,134]. Interestingly, critical for surface localization of CD47 protein is the long 3′-untranslated region (3′-UTR) to which human antigen R (HuR) binds. This commences a chain of reactions that consequently result in plasma membrane translocation of CD47 [137]. Furthermore, after expression of CD47 protein, transcriptional modifications in the form of pyroglutamate formation are needed and which is performed by glutaminyl-peptide cyclotransferase-like (QPCTL). The protein modified in such a way gains the quality of enhanced binding of SIRPα and therefore, inhibits the cancer cell clearance performed by phagocytes [138]. Piecing it all together due to versatile factors that have an impact on CD47 expression, there are many possible approaches to blocking its function in cancer cells. Some of them remain yet undiscovered; however, some have been already studied in possible application in immunotherapy of breast and ovarian cancer. 

#### 3.3.3. CD47 Blockade

Since CD47-SIRPα axis inhibits phagocytosis and, consequently, clearance of cancer cells, effective blockade of this pathway would allow cells like macrophages or dendritic cells to regain their proper function. Moreover, this would reinforce the efficacy of cancer immunotherapy as well [117]. Therefore by blocking CD47 protein, tumor regression could be attained [139,140,141]. It has been observed that CD47-SIRPα axis blockade may be accomplished with use of anti-CD47 antibody [128,139,140,142,143,144]. A similar result was observed with KWAR23—SIRPα specific monoclonal antibody that disrupted SIRPα-CD47 interaction [145]. Considering the clinical trials, which are currently exploring anti-CD47 antibodies against ovarian cancer, they aim to block this signal and enhance the immune system’s ability to eliminate cancer cells, offering a promising therapeutic strategy [123,146].

#### 3.3.4. Targeting CD47-SIRPα Axis in Breast Cancer Immunotherapy 

A study was conducted on mice that had cancer induced with medroxyprogesterone acetate (MPA) and DNA-damaging agent dimethylbenzantracene (DMBA). Moreover, the cancer cells presented with significant expression of CD47 protein on their surface [147]. Therefore, a therapy of systemic ani-CD47 antibody injections was performed either by proceeding with four injections or five times per week throughout the duration of the experiment which lasted 60 days. It was discovered that both therapy schedules resulted in reduced tumor growth and they significantly prolonged survival of the mice who had carcinogen-induced breast cancer [147]. Furthermore, combining CD47 blockade (administered every day since diagnosis) with the anthracycline mitoxantrone (MTX) (administered once, upon the diagnosis of the tumor) showed that the attained reduction in tumor growth was even more significant than when these two types of treatment were used separately. When the combination of CD47-directed immunotherapy with MTX-based chemotherapy was studied on AT3 cancers, no additive interaction of these drugs resulting in tumor growth blockade was observed [147]. However, injected antibodies during the combined therapy caused depletion of CD4+ and CD8+ lymphocytes which allowed the elimination of tumor growth. In order to further explore that revelation, the composition of immune infiltrate of AT3 influenced by CD47 blockage was studied. CD47 blockade resulted in elimination of immunosuppressive cells from the tumor microenvironment, consequently favoring an anticancer immune response [147].

Another application of targeting CD47 protein in breast cancer therapy may be found by eliminating aggressive radioresistant cancer cells [148]. It was revealed that the aggressive phenotype of radioresistant BC cells is associated with expression of two receptors—HER2 and CD47. Interestingly HER2-expressing cells tend to present with upregulated CD47 protein as well. Therefore an integrated therapeutic approach targeting blockade of both CD47 and HER2 is needed in order possibly to abolish resistant cancer cells in radiotherapy of breast cancer [148]. Trastuzumab, a targeted anti-HER2 monoclonal antibody, is highly effective in the treatment of early-stage HER2+ breast cancer. However, despite persistence of HER2 gene amplification or overexpression, the majority of patients who at first positively respond to trastuzumab treatment develop resistance to treatment and relapse [149]. The important mechanism standing behind it is based upon upregulation of CD47 protein [149,150]. The aim of Upton et al. was to leverage HER2 overexpression to engage antibody-dependent cellular phagocytosis (ADCP). That was to be attained with combination of trastuzumab and blockade of CD47 with Hu5F9-G4 (Magrolimab), a humanized monoclonal antibody against CD47 which facilitates macrophage-mediated phagocytosis. It was discovered that combining trastuzumab with anti-CD47 potentiated antibody-dependent cellular phagocytosis of human HER2+ breast cancer cells [149]. Therefore, implementing anti-CD47 antibodies to anti-HER2 treatment of HER2+ breast cancer may be beneficial in treating those patients whose cancers have progressed after anti-HER2 therapy. The suggested treatment can augment anti-tumor efficacy in HER2+ breast cancer even if the patient has not developed clinical trastuzumab resistance. In that way, the likelihood of relapse caused by resistance to that drug may be reduced. Apart from overcoming trastuzumab resistance, enhanced ADCP mediated by Magrolimab has the potential of activating tumor-specific anticancer responses because of augmented cross-presentation, therefore increasing tumor immunosurveillance whilst decreasing immune evasion of cancer cells [149]. Moreover, blockade of CD47-SIRPα axis results in converting tumor associated macrophages (TAMs) into an anti-tumor state and consequently provides augmented phagocytosis and suppressed growth of the tumor [149,151,152,153]. 

#### 3.3.5. Targeting CD47-SIRPα Axis in Ovarian Cancer Immunotherapy 

Various approaches in treatment of ovarian cancer (OC) by targeting CD47-SIRPα axis are being researched in great numbers. One of them is blocking the CD47-SIRPα axis in monotherapy [146]. Anti-CD47 monoclonal antibodies (B6H12.2 and BRIC126) were found to promote human and mouse phagocytosis of SK-OV-3 OC cells in vitro, respectively [128]. In murine models they inhibited tumor growth and increased the survival of the mice over time [154]. In a different study, anti-CD47 antibody (B6H12.2) increased macrophage infiltration in xenograft ovarian cancer cells and induced significant phagocytosis of OC stem cells [155,156]. Interestingly, these cells are known for their proliferation, recurrence, and resistance; hence, it has been suggested that anti-CD47 monoclonal antibodies may play a significant part in prevention and treatment of metastatic and recurrent ovarian cancer [146,155,156]. Applying anti-CD47 mAbs in TOV OC cell lines resulted in inhibition of tumor cell growth—their migration in invasion [157]. A selective humanized-IgG4 mAb—BI 765063, acts as SIRPα antagonist. When administered to nine patients with OC during a phase I study it was well tolerated, no dose-limited toxicities or hemotoxic adverse drug reactions were reported, and it presented well with regard to pharmacokinetics and efficacy [146,154]. Even though the course of blocking CD47-SIRPα axis in monotherapy has shown a remarkable potential, applying combination strategies that would inhibit this pathway may be even more promising [146]. That is because inhibition of CD47-SIRPα signaling has been shown to restore concurrently macrophage phagocytosis and activate T cells, thus combining innate and adaptive immune checkpoint inhibitors (ICIs) to bring promising results [129,140,158]. Since specific targets are lacking for ovarian cancer, targeting PD-1/PD-L1 therapies remains a field of research [146]. By combining anti-CD47 mAb with ani-PD-L1 mAb, the promotion of tumor cell phagocytosis in vitro was obtained as well as suppression of growth of the tumor observed in vivo, which showed the synergy between innate and adaptive ICIs [159]. Even more efficacy and precision is hoped for with administration of bispecific antibodies (BsAbs) [146]. Their skeleton is built of two arms—one blocks the CD47-SIRPα axis and the other binds tumor-specific antigens, thereby increasing the precision of BsAbs. In treating ovarian cancer, the targets for PF-0725787, the dual checkpoint inhibiting BsAb, were CD47/SIRPα and PD-1/PD-L1 in order to maximize anti-tumor immunity [146]. In the tumor microenvironment, activation of dendritic cells and macrophages as well as increased infiltration of CD8+ T cells were observed. Patients with OC, who took part in the study of PF-0725787 in a phase I, presented toleration for priming and subsequent dose [160]. Another application of immunotherapy of OC might be based on the combination of chemotherapy and anti-CD47 antibody [161,162]. Indeed, such combination of chemotherapy and photodynamic therapy drugs with anti-CD47 antibody, specifically its Fab segment has been researched. It was established that such a course of treatment may improve the reactivity of OC OVCAR-3 cells to drugs [163]. Dual CAR-T cells were generated by Shu et al. to co-target CD-47 and TAG-72, an aberrantly glycosylated glycoprotein overexpressed in adenocarcinomas such as ovarian cancer. These CAR-T cells presented effective elimination of OVCAR-3 cells in vitro which suppressed tumor growth in OC xenografted mice. Moreover, specific targeting of TAG-72 enabled reduction of normal tissue which is a highly desired quality [164]. A distinct theory is based on using oncolytic virus (OV) to infiltrate drugs into the microenvironment of the tumor. Such a virus, OV-αCD47, was indeed constructed to deliver anti-CD47 mAb. OC-engrafted mice administration of the virus, especially the one with the IgG1 skeleton, resulted in enhanced innate immunity, presented with oncolytic function, and consequently prolonged survival in the mice [165].

## 4. Summary 

Cancer cells create a unique environment which is highly different from that which we observe normally in the human body. The tumor microenvironment consists of low nutrient supply, hypoxia, inflammation, and many others. At the same time there are more phenotypes of immune cells than there are in healthy tissue. To induce such specialized setting tumor cells utilize immunological pathways which can be specific to those cells. 

Signal transducer and activator of transcription 3 (STAT3) is a molecule which plays a crucial role in regulating the cellular process. Activation of STAT3 in cancer cells leads to proliferation and suppression of apoptosis and we can observe that in BC where STAT3 is activated by elevated levels of IL-6 and IL-10. Additionally, STAT3 promotes activation of osteoblasts which enhances the probability of metastasis to the bones. STAT3 together with IL-6 can also lead to activation of epithelial to mesenchymal transition in BC and cervical carcinoma. ANXA1 is a protein involved in membrane-related effects and it has been shown that it regulates immune response, cell proliferation, and apoptosis. The level of this protein is connected with posttranslational modifications of proteins and because of that its expression varies in accordance with tumor type. ANXA1 is connected with antiproliferative properties by modulating EGF and EGFR tyrosine kinase. It can also mediate apoptosis by dephosphorization of BAD or by migrating to the cell surface and binding phosphatidylserine. On the one hand loosing ANXA1 would be beneficial for cancer cells because it could lead to resistance to apoptosis but on the other hand the presence of ANXA1 during apoptosis creates peripheral immune tolerance of cancer antigens by Dectin-1 receptor which helps the cancer being unnoticed by the immune system. Studies show that when ANXA1 is absent, tumor growth is slower, it forms fewer blood vessels, and its metastatic potential is lower. In breast cancer, the level of ANXA1 can vary but regardless of the level of this molecule the correlation between the concentration of ANXA1 and the resistance to chemotherapy was not found. It was also suggested that ANXA1 can operate as tumor suppressor gene because it can modulate proliferative function of estrogens. The effect of high expression of ANXA1 in BC is highly connected with the type of BC present; however, in invasive basal-like breast cancer, triple negative breast cancer, familial breast cancer with BRCA1, and BRCA 2 high levels of ANXA1 were connected with poorer prognosis. Cluster of differentiation (CD47) is transmembrane glycoprotein which can be found in all types of cells. When its domain connects with signal regulatory protein α (SIRPα), which is present on macrophages, monocytes, dendric cells, and neurons, the phosphorylation of SIRPα takes place which is known as “do not eat me signal”. That means that one of the main roles of CD47 is blocking phagocytosis which can lead to cancer cells being undetected by macrophages. However, CD47 can be also found on red blood cells which can lead to splenomegaly when targeted therapy against CD47 is used. High expression of CD47 can be observed on ovarian cancer, breast cancer, squamous cell carcinoma of the head and neck, gliomas, osteosarcoma, melanoma, glioblastoma, bladder cancer, colon cancer, hepatocellular cancer, and in myeloid leukemia stem cells. There are several factors that lead to upregulation of CD47: Myc oncogene, TNFα which activates NfκB, hypoxia together with expression of HIF-1; all of them lead to higher expression of CD47 on the cell surface which causes worse prognosis for patients. Additionally, CD47 can be spread by exosomes which are actively secreted and can enter other cells. We can use this knowledge and create therapy which will block CD47 on cancer cells and by this we can reactivate proper function of macrophages and dendric cells. To disable CD47, we can use anti-CD47 antibodies or KWAR23–SIRPα specific monoclonal antibody. It has been observed that usage of anti-CD47 antibodies on mice with breast cancer substantially prolonged survival of the animals. This form of therapy can also be used on HER2 radioresistant BC because in this type of cancer the CD47 receptor is highly upregulated. When anti-CD47 antibodies were administered, antibody-dependent phagocytosis was activated as well as TAMs being converted into an anti-tumor state which lead to suppression of tumor. Blockage of CD47 shows also very promising results in therapy of ovarian cancer. Monotherapy with anti-CD47 antibodies leads to inhibition of tumor growth in SK-OV-3 OC cells and inhibition of growth, migration, and invasion of TOV OC. Even better results were achieved when blockage of CD47 was connected with blockage of PD-L1. It was achieved by creating bispecific antibodies which when administered lead to activation of dendric cells and increased infiltration of CD8+ cells in the tumor environment.

Our knowledge of oncogenic pathways allows us to create targeted treatments which are not only highly effective but also are highly specific, which is a very desired quality because it enables us to eliminate the high number of side effects associated with traditional chemotherapy. However, most of the research is conducted in vitro or on animal models which does not give us confidence on how such therapy will work in humans. Because of that, we still need to continue to work in order to better understand the nature of tumor microbiology and how this affects the human body.

## Figures and Tables

**Table 1:** Selected drugs targeting elements of TME of BC in preclinical and clinical studies [19,20,21,22,23,24,25,26,27].

Drugs	Mechanism	Current Status	Refs.
Sibrotuzumab	Anti-fibroblast activation protein (Anti-FAP) labeled with radioisotope 131I that prevents FAP from degrading ECM proteins	Phase I	[22]
Tetrathiomolybdate	Copper chelator that targets lysyl oxidase (LOX) and thus affects copper-dependent components of TME	Phase II	[23,24,25]
Sotigalimab	Antibodies agonistic towards CD40, which alongside with its ligand can restore T cell anti-tumor activity	Phase II	[19,20,21]
Reparixin	Chemokine receptors 1 and 2 (CXCR1/2) antagonist that demonstrates activity against BC stem cells (BCSCs) in BC	Phase I/II	[26,27]

**Table 2:** Variety of roles of ANXA1 in different BC types [106,107,108,109,110,111,112].

ANXA1 Role in Cancer	Cancer Type	Ref.
ANXA1 expression is associated with a highly invasive basal-like BC subtype both in a panel of human breast cancer cell lines as in breast cancer patients and that ANXA1 is related to breast cancer progression, promotes metastasis by enhancing TGF beta/Smad signaling and actin reorganization. ANXA1 knockdown in IBL cancer cells reduced the number of spontaneous lung metastasis. Additional expression of ANXA1 enhances metastatic spread.	invasive basal-like (IBL) breast cancer	[106]
Tissue microarrays of BC samples observed a higher expression of AnxA1 in TNBCs and in lymph node metastasis. Positive correlation in primary tumors between expression levels of ANXA1 and its receptor, FPR1. Despite displaying a lesser strength, this correlation also exists in BC lymph node metastasis; AnxA1 is highly expressed and secreted in the TNBC cell line MDA-MB-231 that also expressed high levels of FPR1.	triple-negative breast cancer (TNBC)	[107]
ANXA1 can enhance the function of Treg cells and reduce the survival rate of patients with breast cancer. Targeting ANXA1 can reduce Treg cell function and shrink breast tumors.	triple-negative breast cancer	[108]
ANXA1 high tumors are associated with activated mast cells and M2 macrophages (*p* > 0.01), but do not show any association with tumor heterogeneity nor cytolytic activity. High expression of ANXA1 group enriched inflammation, epithelial-to-mesenchymal transition (EMT), and angiogenesis-related genes in a gene set enrichment assay in both cohorts.	triple-negative breast cancer	[109]
In MCF-7 cells, ANXA1-targeting small interfering RNA (siRNA) reduced ANXA1 mRNA and protein levels and attenuated cell proliferation induced by FCS, estradiol, or epidermal growth factor. In invasive breast cancer, immunohistochemistry revealed the presence of ANXA1 and its receptor, FPR2, in both tumor epithelium and stromal cells. These observations suggest a novel signaling role for ANXA1 in mitogen-activated proliferation of breast tumor epithelial cells that is mediated via activation of FPR1 and FPR2.	estrogen receptor (ER)-positive MCF-7 and ER-negative MDA-MB-231 breast tumor cell lines	[110]
The frequency of ANXA1 positive tumors was higher in familial breast cancer patients with BRCA1/2 mutations than in BCAC patients, with 48.6% versus 12.4%, respectively; *p* < 0.0001. ANXA1 was also highly expressed in BCAC tumors that were poorly differentiated, triple negative, EGFR-CK5/6 positive or had developed in patients at a young age. ANXA1 was a significant independent predictor of survival in HER2+ patients (10-years BCSS:HRadj = 1.70; 95% CI = 1.17–2.45). ANXA1 is overexpressed in familial breast cancer patients with BRCA1/2 mutations and correlated with poor prognosis features: triple negative, and poorly differentiated tumors. ANXA1 might be a biomarker candidate for breast cancer survival prediction in high-risk groups such as HER2+ cases.	triple negative, EGFR-CK5/6 positive familial breast cancer patients with BRCA1/2	[111]
The expression of FOXO1, GATA3 and Annexin-1 were all inversely correlated with lymph node-positive tumors. Both FOXO1 and Annexin-1 expression were also inversely associated with HER2-overexpressing tumors. ANXA1 levels independently do not predict DFS.	HER2-overexpressing tumors	[112]
